# Supplementary material for: Barriers and Facilitators of Safe Communication in Obstetrics: Results from Qualitative Interviews with Physicians, Midwives and Nurses
Source: Int J Environ Res Public Health. 2021 Jan 21;18(3):915. doi: 10.3390/ijerph18030915 (PMC7908168; doi:10.3390/ijerph18030915)
Supplement: Supplementary file 1 [file ijerph-18-00915-s001.pdf]

Supplementary

Table S1: Subjective theories about causes of pAE and suggestions to avoid pAE

Team Level		
**Midwives**	**Physicians**	**Nurses**
**Communication**		
The most important thing is definitely always that someone speaks it out loud and someone takes over the communication and says 'They do this, you do that, he does that' (P2Midwife).<br><br>The most important thing in obstetrics during stressful situations is to stay calm, so the doctors or midwives who get hectic and irritable and try to do everything at the same time, communication gets lost and you often misunderstand each other and the doctors and midwives who stay calm, look into each other's eyes and say 'I've seen this and that, what do you think about it', it works extremely well (P3Midwife).	It was not understood rightly which drug was meant, because it was somehow called differently in the country (of origin of the colleague) and then another drug was given (P4Physician).<br><br>So I think what is very important is that you are not afraid to appear stupid, so that I as a resident can always ask my senior physicians, even if I feel I should be able to assess and decide it, but somehow I can't. That even in a situation where I feel stupid, just say that it's about the safety of the patient or the baby or whatever, I'll just ask my senior physician and I think you can apply that to all combinations between the professions (P15Physician).	(…) a woman should not breastfeed because of her five medications, but our doctor on the ward did not know that, neither did we (the nurses); the mother told herself the pediatrician (...). The mother has now been breastfeeding for two days, it is not dangerous, but she is sad and disappointed (P11Nurse).<br><br>When we get patients, I take care of the child, I go to mother, (…) and then I see if it's freshly made or not, that's not an issue, but then I expect my colleague, who looked after her for four hours in the delivery room, to tell me 'I didn't make it, you do it' (P11Nurse).
**Documentation**		
**Midwives**	**Physicians**	**Nurses**
Sometimes the fever curve is not readjusting so quickly when one gets a new medication, or a different dosage, and that's just what the doctors tell a midwife, and the midwife just says 'yes, I'll tell the colleague who is responsible', but maybe she'll forget about it, or something (P20Midwife).	We have completely separate handovers, so the doctors talk to each other, the nurses talk to each other and the midwives talk to each other, (...); probably it is not wrong, at least as far as the delivery room is concerned, that when the doctors perform the handover, a midwife is present, who can take the information with her, and vice versa; when the midwives do the handover, one of the doctors is present (P15Physician).	And you often cannot read the hand writing, and then it is sometimes dangerous for me. I just have to search, and that takes a lot of time. And I think the whole maternity ward and gynecology department is always searching for missing files (P16 Nurse).

Clinic/ward level		
**Midwives**	**Physicians**	**Nurses**
…if we train certain emergencies as a team in some simulations, I don't think that would completely resolve the situation, but it might minimize those risky situations (P20Midwife).<br><br>(...) an offer, if a course of birth was frustrating, somebody you can later on talk about it; maybe with a doctor who can teach you in a medical way and say 'ok, this indication was given in any case' to an emergency cesarean section or something (P2Midwife).	(...) if you didn't have the pressure to manage your ward and the delivery room and the consultation hours at the same time and the telephone didn't always ring, then not quite as much would slip through your fingers and you would perhaps notice more of the women, simply five minutes more time per patient, (…) I think that would make a big difference (P4Physician).<br><br>Actually some support for the staff who do not speak German so well. So that they could learn German more easily, or could learn German better before they start, if that is possible (P10Physician).	I believe that bed management would be a quite good thing. And what I would see very positively is an admission and discharge service. I think it would bring a lot of calm (P16Nurse).<br><br>A kind of supervision, maybe for certain cases. Quickly. It is sometimes when I say 'come on, this is for the case consultation' yes, but that was the day before yesterday and now it lasts six weeks (P8Nurse).
**Health Care System Level**		
**Midwives**	**Physicians**	**Nurses**
…that I don't have to clear out the storage and things like that (...); that I can concentrate on my midwifery work, because I didn't become a midwife to sit at the computer for five hours out of the eight hours shift (P19Midwife)!	More staff. This is a recurring topic. The midwives, they have far too many patients per midwife who have to be cared for. They're also called in at every point and have to do something. And from our side, too, which often makes us so stressed that we don't take the time, I think, or that we simply often get lost (P18Physician)!	(...) so usually there is more work now, more patients, less staff, because there is more written stuff, less time for the patient – patient care used to be more individual, now is almost fifty-fifty, more writing and documentation, every year grows new documentation strain (P11Nurse)!
